# The Baltimore Medical College

**Published:** 1881-09

**Authors:** 


THE BALTIMORE MEDICAL COLLEGE.—This *new* organization expects to open its first session in October, proximo. It is said to be "founded upon Christianity and science," and other new features are the "admission of both sexes as students, and the establishment of a chair of dentistry as a special branch of medicine. Students in dentistry will thus be graduated with the degree of M.D., instead of D.D.S. The course is to be from seven to nine months." President, Prof. Harvey L. Byrd, M.D., Dean, Prof. W. Robert Monroe, M.D.; Prof. B. F. Leonard, M. D., Prof. Henry Froehling, M. D., Prof. L. Roberts Coates, M.D., Prof. L. Wheaton Clapp, M. D. Other professors are to be elected in a few days.

